# Supplementary material for: Analysis of PPARγ Signaling Activity in Psoriasis
Source: Int J Mol Sci. 2021 Aug 10;22(16):8603. doi: 10.3390/ijms22168603 (PMC8395241; doi:10.3390/ijms22168603)
Supplement: Supplementary file 1 [file ijms-22-08603-s001.zip › Supplemental materials_Analysis of PPARg signaling activity in psoriasis/Pathway models/Models images and html files/Model of PPARG signaling in psoriasis/8373.html]

OLR1


# Protein OLR1

|  |  |
| --- | --- |
| URN | urn:agi-llid:4973 |
| Total Entities | 0 |
| Connectivity | 1631 |
| Name | OLR1 |
| Description | oxidized low density lipoprotein receptor 1 |
| Notes | This gene encodes a low density lipoprotein receptor that belongs to the C-type lectin superfamily. This gene is regulated through the cyclic AMP signaling pathway. The encoded protein binds, internalizes and degrades oxidized low-density lipoprotein. This protein may be involved in the regulation of Fas-induced apoptosis. This protein may play a role as a scavenger receptor. Mutations of this gene have been associated with atherosclerosis, risk of myocardial infarction, and may modify the risk of Alzheimer's disease. Alternate splicing results in multiple transcript variants.[provided by RefSeq, Feb 2010] |
| Primary Cell Localization | Plasma membrane |
| Class | Receptor |

---

|  |  |
| --- | --- |
| Pathway | Scavenger Receptors in Platelet Aggregation |
|  | Proteins Involved in Atherosclerosis |
|  | Scavenger Receptor OLR1 in Inflammation Related Endothelial Dysfunction |
|  | Proteins Involved in Diabetic Neuropathy |
|  | Proteins Involved in Age-Related Macular Degeneration |
|  | Noradrenaline/Gs Expression Targets |
|  | Noreadrenaline/Gq Expression Targets |
|  | Elevated Receptors -> Expression Targets in Aorta |
|  | F2 -> AP-1/CREB/ELK/SRF/SP1 Expression Targets |
|  | F2 -> STAT1/NF-kB Expression Targets |
|  | HBEGF -> AP-1/ATF Expression Targets |
|  | Oxidized LDL/OLR1 Activates Cell Proliferation in Cancer |
|  | IL6 Expression Targets |
|  | IL4 Expression Targets |
|  | EDN1 Expression Targets |
|  | AGT -> CREB Expression Targets |
|  | AGT -> STAT Expression Targets |
|  | TGFB1-TGFBR2 Expression Targets |
|  | TGFB1-TGFBR1/AP-1 Expression Targets |
|  | TGFB1-ACVRL1 Expression Targets |
|  | TNF -> NF-kB Expression Targets |
|  | TNF -> AP-1 Expression Targets |
|  | IL1B Expression Targets |
|  | TLR4 -> NF-kB/IRF Expression Targets |
|  | TLR9 Expression Targets |
|  | Model of PPARG signaling in psoriasis |
|  | PPARG negative regulators and targets |
|  | 2\_Oxidized LDL causes endothelial dysfunction in atherosclerosis |

---

|  |  |
| --- | --- |
| MedScan ID | 4973 |

---

|  |  |
| --- | --- |
| LocusLink ID | 4973 |
|  | 108078 |
|  | 140914 |

---

|  |  |
| --- | --- |
| Alias | Lectin-like oxidized low-density lipoprotein receptor-1 |
|  | hLOX-1 |
|  | lectin-like oxidized low-density lipoprotein receptor |
|  | LOX-1 |
|  | LOX-I |
|  | oxidized low density lipoprotein (lectin-like) receptor 1 |
|  | LOXIN |
|  | oxidized low density lipoprotein (lectin-like) receptor I |
|  | SCARE1 |
|  | oxidized low-density lipoprotein receptor 1 |
|  | oxidized LDL receptor 1 |
|  | Oldlr1 |
|  | OLR1 |
|  | lectin-type oxidized LDL receptor I |
|  | scavenger receptor class E, member 1 |
|  | oxidized low-density lipoprotein receptor 1, soluble form |
|  | SLOX1 |
|  | scavenger receptor class E |
|  | Oldr1 |
|  | SR-EI |
|  | CLEC8A |
|  | Lectin-like OXIDIZED LDL receptors |
|  | lectin-like oxLDL receptor 1 |
|  | scavenger receptor class E, member I |
|  | lectin-type oxidized LDL receptor 1 |
|  | lectin-like oxidized LDL receptor 1 |
|  | C-type lectin domain family 8 member A |
|  | ox LDL receptor 1 |
|  | Lectin-like OXIDIZED LDL receptor |
|  | LOX1 |
|  | ox-LDL receptor 1 |
|  | oxidised low density lipoprotein (lectin-like) receptor 1 |
|  | oxidised low density lipoprotein (lectin-like) receptor I |

---

|  |  |
| --- | --- |
| GO ID | 0030246 |
|  | 0008015 |
|  | 0007155 |
|  | 0006954 |
|  | 0050900 |
|  | 0043312 |
|  | 0006508 |
|  | 0005576 |
|  | 0005887 |
|  | 0043231 |
|  | 0016020 |
|  | 0045121 |
|  | 0005654 |
|  | 0005886 |
|  | 0043235 |
|  | 0035579 |
|  | 0070821 |
|  | 0005041 |
|  | 0008219 |
|  | 0002376 |
|  | 0007159 |
|  | 0042157 |
|  | 0010629 |
|  | 0019897 |
|  | 0016021 |
|  | 0097756 |
|  | 0032930 |
|  | 0042542 |
|  | 0016192 |
|  | 0006898 |
|  | 0070062 |
|  | 0004872 |
|  | 0007596 |
|  | 0005634 |

---

|  |  |
| --- | --- |
| KEGG ID | hsa:4973 |
|  | mmu:108078 |
|  | rno:140914 |

---

|  |  |
| --- | --- |
| Organism | Homo sapiens {Organism urn:agi-taxid:9606} |
|  | Mus musculus {Organism urn:agi-taxid:10090} |
|  | Rattus norvegicus {Organism urn:agi-taxid:10116} |
|  | Homo sapiens |
|  | Mus musculus |
|  | Rattus norvegicus |

---

|  |  |
| --- | --- |
| Mouse chromosome position | 6 |

---

|  |  |
| --- | --- |
| OMIM ID | 602601 |
|  | 608446 |

---

|  |  |
| --- | --- |
| Rat chromosome position | 4q42 |

---

|  |  |
| --- | --- |
| Hugo ID | 8133 |
|  | HGNC:8133 |

---

|  |  |
| --- | --- |
| Human chromosome position | 12p13.2 |
|  | 12p13.2-p12.3 |

---

|  |  |
| --- | --- |
| Swiss-Prot Accession | P78380 |
|  | A0A024RAU0 |
|  | P78380.1 |
|  | G4WK10 |
|  | G4WK09 |
|  | Q9EQ09 |
|  | Q9EQ09.2 |
|  | A0A0G2K1K0 |
|  | O70156 |
|  | O70156.1 |
|  | A8K7V9 |
|  | B4DI48 |
|  | G3V1I4 |
|  | Q2PP00 |
|  | Q7Z484 |
|  | Q3U3M1 |

---

|  |  |
| --- | --- |
| PIR ID | JE0111 |

---

|  |  |
| --- | --- |
| GenBank ID | NC\_000012 |
|  | NM\_001172632 |
|  | NP\_001166103 |
|  | NM\_001172633 |
|  | NP\_001166104 |
|  | NM\_002543 |
|  | NP\_002534 |
|  | NG\_016743 |
|  | AC024224 |
|  | AC115676 |
|  | AH007081 |
|  | AAC97927 |
|  | AJ131757 |
|  | CAB38175 |
|  | AX429256 |
|  | CAD36117 |
|  | CH471094 |
|  | EAW96156 |
|  | EAW96157 |
|  | EAW96158 |
|  | EAW96159 |
|  | CS443020 |
|  | CAL68889 |
|  | DQ314885 |
|  | ABC40744 |
|  | JA885104 |
|  | CCQ77677 |
|  | AB010710 |
|  | BAA24580 |
|  | AB102861 |
|  | BAC81565 |
|  | AF035776 |
|  | AAC82329 |
|  | AK292124 |
|  | BAF84813 |
|  | AK295409 |
|  | BAG58360 |
|  | AK298040 |
|  | BAG60339 |
|  | AK308009 |
|  | AK316353 |
|  | BAH14724 |
|  | AL551100 |
|  | AX429248 |
|  | CAD36113 |
|  | BC022295 |
|  | AAH22295 |
|  | BC055085 |
|  | BP219342 |
|  | BQ028409 |
|  | CA309323 |
|  | CB955704 |
|  | P78380 |
|  | NC\_000072 |
|  | XR\_001785081 |
|  | XM\_017321299 |
|  | XP\_017176788 |
|  | XM\_017321300 |
|  | XP\_017176789 |
|  | NM\_001301096 |
|  | NP\_001288025 |
|  | NM\_001301094 |
|  | NP\_001288023 |
|  | NM\_138648 |
|  | NP\_619589 |
|  | AC161602 |
|  | CH466523 |
|  | EDK99928 |
|  | AF303744 |
|  | AAG44998 |
|  | AK154687 |
|  | BAE32764 |
|  | AK160919 |
|  | BX513847 |
|  | HQ603085 |
|  | AEQ16487 |
|  | HQ603086 |
|  | AEQ16488 |
|  | Q9EQ09 |
|  | NC\_005103 |
|  | NM\_133306 |
|  | NP\_579840 |
|  | AC\_000072 |
|  | AABR07073062 |
|  | AAHX01033064 |
|  | AAHX01033065 |
|  | AB018104 |
|  | BAA35123 |
|  | AC115156 |
|  | CH473964 |
|  | EDM01737 |
|  | EDM01738 |
|  | AB005900 |
|  | BAA25785 |
|  | BC097290 |
|  | AAH97290 |
|  | FQ220710 |
|  | O70156 |
|  | XM\_011520682 |
|  | XP\_011518984 |
|  | XM\_011520683 |
|  | XP\_011518985 |
|  | NC\_018923 |
|  | AF079164 |
|  | AF079165 |
|  | AF079166 |
|  | AF079167 |
|  | AMYH02026023 |
|  | DQ891409 |
|  | ABM82335 |
|  | DQ894585 |
|  | ABM85511 |
|  | AC\_000028 |
|  | AAHY01057559 |
|  | AAHY01057560 |
|  | AAHY01057561 |
|  | AAHY01057562 |
|  | BC148550 |
|  | AAI48551 |
|  | XM\_006719081 |
|  | XP\_006719144 |
|  | AC\_000144 |
|  | ABBA01067796 |
|  | XM\_006505303 |
|  | XP\_006505366 |
|  | XM\_006505304 |
|  | XP\_006505367 |

---

|  |  |
| --- | --- |
| Swiss-Prot ID | OLR1\_HUMAN |
|  | OLR1\_MOUSE |
|  | OLR1\_RAT |

---

|  |  |
| --- | --- |
| Cell Localization | Cell membrane |
|  | Membrane raft |
|  | Secreted |

---

|  |  |
| --- | --- |
| Ensembl ID | ENSG00000173391 |
|  | ENSP00000405116.2 |
|  | ENST00000432556.6 |
|  | ENSP00000439251.1 |
|  | ENST00000545927.5 |
|  | ENSP00000309124.3 |
|  | ENST00000309539.8 |
|  | ENSMUSG00000030162 |
|  | ENSMUSP00000138588.1 |
|  | ENSMUST00000182784.3 |
|  | ENSMUSP00000138228.1 |
|  | ENSMUST00000183258.7 |
|  | ENSMUSP00000032265.6 |
|  | ENSMUST00000032265.12 |
|  | ENSRNOG00000056219 |
|  | ENSRNOP00000071872.1 |
|  | ENSRNOT00000086390.1 |
|  | ENSP00000405116 |
|  | ENST00000432556 |
|  | ENSP00000439251 |
|  | ENST00000545927 |
|  | ENSP00000309124 |
|  | ENST00000309539 |
|  | ENSMUSP00000138588 |
|  | ENSMUST00000182784 |
|  | ENSMUSP00000138228 |
|  | ENSMUST00000183258 |
|  | ENSMUSP00000032265 |
|  | ENSMUST00000032265 |
|  | ENSRNOP00000071872 |
|  | ENSRNOT00000086390 |
|  | ENSRNOG00000008375 |
|  | ENSRNOP00000011196 |
|  | ENSRNOT00000011196 |

---

|  |  |
| --- | --- |
| MGI ID | MGI:1261434 |

---

|  |  |
| --- | --- |
| RGD ID | 620515 |

---

|  |  |
| --- | --- |
| Unigene ID | Hs.412484 |
|  | Mm.293626 |
|  | Rn.87449 |

---

|  |  |
| --- | --- |
| Homologene ID | 1910 |

---

|  |  |
| --- | --- |
| Shape | Stick-vertex |

---
